# *N*-(1,5-Dimethyl-3-oxo-2-phenyl-2,3-di­hydro-1*H*-pyrazol-4-yl)-2-(4-nitro­phen­yl)acetamide

**DOI:** 10.1107/S1600536814009738

**Published:** 2014-05-03

**Authors:** Manpreet Kaur, Jerry P. Jasinski, H. S. Yathirajan, B. Narayana, K. Byrappa

**Affiliations:** a Department of Studies in Chemistry, University of Mysore, Manasagangotri, Mysore 570 006, India; b Department of Chemistry, Keene State College, 229 Main Street, Keene, NH 03435-2001, USA; c Department of Studies in Chemistry, Mangalore University, Mangalagangotri 574 199, India; d Materials Science Center, University of Mysore, Vijyana Bhavan Building, Manasagangothri, Mysore 570 006, India

## Abstract

In the title compound, C_19_H_18_N_4_O_4_, the nitro­phenyl and phenyl rings are twisted by 67.0 (6) and 37.4 (4)°, respectively, with respect to the pyrazole ring plane [maximum deviation = 0.0042 (16) Å]. The dihedral angle between the mean planes of the phenyl rings is 59.3 (3)°. The amide group, with a C—N—C—C torsion angle of 177.54 (13)°, is twisted away from the plane of the pyrazole ring in an anti­periplanar conformation. In the crystal, N—H⋯O hydrogen bonds involving the carbonyl group on the pyrazole ring and the amide group, together with weak C—H⋯O inter­actions forming *R*_2_^2^(10) graph-set motifs, link the mol­ecules into chains along [100]. Additional weak C—H⋯O inter­actions involving the nitro­phenyl rings further link the mol­ecules along [001], also forming *R*_2_^2^(10) graph-set motifs, thereby generating (010) layers.

## Related literature   

For the structural similarity of *N*-substituted 2-aryl­acetamides to the lateral chain of natural benzyl­penicillin, see: Mijin & Marinkovic (2006 ▶); Mijin *et al.* (2008 ▶). For the coordination abilities of amides, see: Wu *et al.* (2008 ▶, 2010 ▶). For the pharmaceutical, insecticidal and non-linear properties of pyrazoles, see: Chandrakantha *et al.* (2013 ▶); Cheng *et al.* (2008 ▶); Hatton *et al.* (1993 ▶); Liu *et al.* (2010 ▶). For related structures, see: Fun *et al.* (2012 ▶); Butcher *et al.* (2013*a* ▶,*b* ▶); Kaur *et al.* (2013 ▶); Mahan *et al.* (2013 ▶). For standard bond lengths, see: Allen *et al.* (1987 ▶).
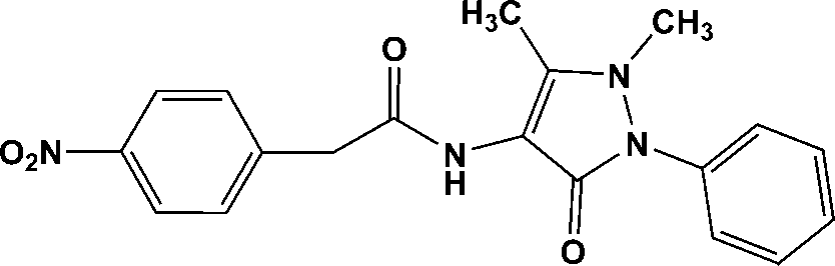


## Experimental   

### 

#### Crystal data  

C_19_H_18_N_4_O_4_
*M* *_r_* = 366.37
Triclinic, 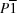

*a* = 6.7023 (6) Å
*b* = 8.6335 (8) Å
*c* = 15.8720 (13) Å
α = 76.305 (7)°
β = 84.399 (7)°
γ = 77.252 (7)°
*V* = 869.33 (13) Å^3^
*Z* = 2
Cu *K*α radiation
μ = 0.84 mm^−1^
*T* = 173 K
0.28 × 0.22 × 0.12 mm

#### Data collection  

Agilent Eos Gemini diffractometer
Absorption correction: multi-scan (*CrysAlis PRO* and *CrysAlis RED*; Agilent, 2012 ▶) *T*_min_ = 0.851, *T*_max_ = 1.000
5113 measured reflections
3262 independent reflections
2913 reflections with *I* > 2σ(*I*)
*R*_int_ = 0.030

#### Refinement  

*R*[*F* ^2^ > 2σ(*F* ^2^)] = 0.046
*wR*(*F* ^2^) = 0.129
*S* = 1.07
3262 reflections
247 parameters
H-atom parameters constrained
Δρ_max_ = 0.27 e Å^−3^
Δρ_min_ = −0.21 e Å^−3^

### 

Data collection: *CrysAlis PRO* (Agilent, 2012 ▶); cell refinement: *CrysAlis PRO*; data reduction: *CrysAlis RED* (Agilent, 2012 ▶); program(s) used to solve structure: *SUPERFLIP* (Palatinus & Chapuis, 2007 ▶); program(s) used to refine structure: *SHELXL2012* (Sheldrick, 2008 ▶); molecular graphics: *OLEX2* (Dolomanov *et al.*, 2009 ▶); software used to prepare material for publication: *OLEX2*.

## Supplementary Material

Crystal structure: contains datablock(s) I. DOI: 10.1107/S1600536814009738/hg5393sup1.cif

Structure factors: contains datablock(s) I. DOI: 10.1107/S1600536814009738/hg5393Isup2.hkl

Supporting information file. DOI: 10.1107/S1600536814009738/hg5393Isup3.cml

CCDC reference: 1000268

Additional supporting information:  crystallographic information; 3D view; checkCIF report

## Figures and Tables

**Table 1:** Hydrogen-bond geometry (Å, °)

*D*—H⋯*A*	*D*—H	H⋯*A*	*D*⋯*A*	*D*—H⋯*A*
N1—H1⋯O2^i^	0.86	2.03	2.8658 (18)	164
C7—H7⋯O4^ii^	0.93	2.54	3.307 (2)	139
C18—H18*B*⋯O2^iii^	0.96	2.56	3.336 (2)	138

Symmetry codes: (i) 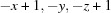; (ii) 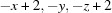; (iii) 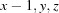.

**Fig. 1.**
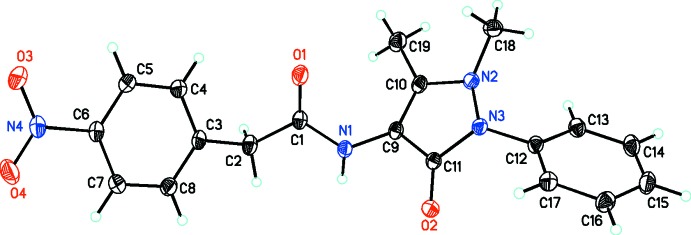
ORTEP drawing of (I) (C19H18N4O4) showing the labeling scheme of the molecule with 30% probability displacement ellipsoids.

**Fig. 2.**
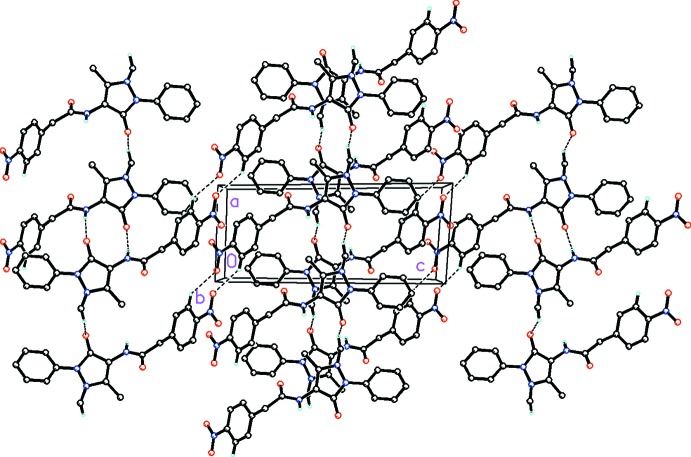
Molecular packing for (I) viewed along the b axis. Dashed lines indicate N—H···O intermolecular hydrogen bonds and weak C—H···O intermolecular interactions forming R22(10) graph set motifs linking the molecules into chains along [100]. Additional weak C—H···O intermolecular interactions involving the nitrophenyl rings link the molecules further along [001] also forming R22(10) graph set motifs viewed with dashed lines. H atoms not involved in hydrogen bonding have been removed for clarity.

**Fig. 3.**
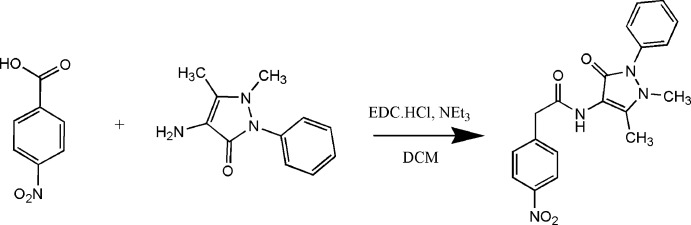
Synthesis scheme of (I).

## supplementary crystallographic information

### 1. Comment

*N*-Substituted 2-arylacetamides are biologically active compounds because of their structural similarity to the lateral chain of natural benzylpenicillin (Mijin *et al.*, 2006, 2008). Amides are also used as ligands due to their excellent coordination abilities (Wu *et al.*, 2008, 2010). In a variety of biological heterocyclic compounds, N-pyrazole derivatives are of great interest because of their chemical and pharmaceutical properties (Cheng *et al.*, 2008). Some of the N-pyrazole derivatives have been found to exhibit good insecticidal activities (Hatton *et al.*, 1993), antifungal activities (Liu *et al.*, 2010) and non-linear optical properties (Chandrakantha *et al.*, 2013). Crystal structures of some related acetamide and pyrazole derivatives including: N-(1,5-dimethyl-3-oxo-2-phenyl-2,3-dihrdro-1H-pyrazol-4-yl)-2- [4-(methylsulfanyl)phenyl]acetamide, (Fun *et al.*, 2012), 2-(2,4-dichlorophenyl)-N-(1,5-dimethyl-3-oxo-2- phenyl)-N-(1,5-dimethyl-3-oxo-2-phenyl-2,3-dihydro-1H-pyrazol- 4-yl)acetamide (Butcher *et al.*, 2013*a*,*b*), 2-(3,4-Dichloro phenyl)-N-(1,5-dimethyl-3-oxo-2-phenyl-2,3-dihydro-1H-pyrazol-4-yl) acteamide (Mahan *et al.*, 2013) and recently N-(1,5-Dimethyl- 3-oxo-2-phenyl-2,3-dihydro-1H-pyrazol-4-yl)-2-phenylacetamide (Kaur *et al.*, 2013) have been reported. In view of the importance of amide derivatives of pyrazoles, this paper reports the crystal structure of the title compound (I), C_19_H_18_N_4_O_4_.

The title compound, (I), C_19_H_18_N_4_O_4_, crystallizes with one independent molecule in the asymmetric unit (Fig. 1). In the molecule, the pyrazole ring is nearly planar with C9 atom showing a maximum deviation of 0.0042 (16)Å. The mean planes of the 4-nitrophenyl and phenyl rings is twisted with respect to that of the pyrazole ring by 67.0 (6)° and 37.4 (4)°, respectively. The dihedral angle between the mean planes of the two phenyl rings is 59.3 (3)°. The amide group, with a torsion angle of 177.54° is twisted away from the plane of the pyrazole ring in an anti-periplanar conformation. Bond lengths are in normal ranges (Allen *et al.*, 1987). Classical N—H···O intermolecular hydrogen bonds involving the pyrazole ring and the amide group along with weak C—H···O intermolecular interactions forming R_2_^2^(10) graph set motifs link the molecules into chains along [100]. Additional weak C—H···O intermolecular interactions involving the nitrophenyl rings link the molecules further along [001] also forming R_2_^2^(10) graph set motifs and further extending crystal packing into a 2-D supramolecular network (Fig. 2).

### 2. Experimental

4-Nitrophenylacetic acid (0.181 g, 1 mmol) and 4-aminoantipyrine (0.203 g, 1 mmol), 1-ethyl-3-(3-dimethylaminopropyl)-carbodiimide hydrochloride (1.0 g, 0.01 mol) and were dissolved in dichloromethane (20 mL). The mixture was stirred in presence of triethylamine at 273 K for about 3 h (Fig. 3). The reaction completion was confirmed by thin layer chormatography. The contents were poured into 100 ml of ice-cold aqueous hydrochloric acid with stirring, which was extracted thrice with dichloromethane. The organic layer was washed with a saturated NaHCO_3_ solution and brine solution, dried and concentrated under reduced pressure to give the title compound, (I). Single crystals were grown from dichloromethane and and further recrystallised from methanol solution by the slow evaporation method which were subsequently used for x-ray studies.

### 3. Refinement

All of the H atoms were placed in their calculated positions and then refined using the riding model with Atom—H lengths of 0.93Å (CH); 0.97Å (CH_2_); 0.96Å (CH_3_) or 0.86Å (NH). Isotropic displacement parameters for these atoms were set to 1.2 (CH, CH_2_, NH)and 1.5 (CH_3_) times *U*_eq_ of the parent atom. Idealised Me refined as rotating group.

### Crystal data

**Table:**

C_19_H_18_N_4_O_4_	*Z* = 2
*M**_r_* = 366.37	*F*(000) = 384
Triclinic, *P*1	*D*_x_ = 1.400 Mg m^−^^3^
*a* = 6.7023 (6) Å	Cu *K*α radiation, λ = 1.54184 Å
*b* = 8.6335 (8) Å	Cell parameters from 2476 reflections
*c* = 15.8720 (13) Å	θ = 5.4–71.4°
α = 76.305 (7)°	µ = 0.84 mm^−^^1^
β = 84.399 (7)°	*T* = 173 K
γ = 77.252 (7)°	Block, colourless
*V* = 869.33 (13) Å^3^	0.28 × 0.22 × 0.12 mm

### Data collection

**Table:**

Agilent Eos Gemini diffractometer	3262 independent reflections
Radiation source: Enhance (Cu) X-ray Source	2913 reflections with *I* > 2σ(*I*)
Detector resolution: 16.0416 pixels mm^-1^	*R*_int_ = 0.030
ω scans	θ_max_ = 71.6°, θ_min_ = 5.4°
Absorption correction: multi-scan (*CrysAlis PRO* and *CrysAlis RED*; Agilent, 2012)	*h* = −4→8
*T*_min_ = 0.851, *T*_max_ = 1.000	*k* = −10→10
5113 measured reflections	*l* = −19→19

### Refinement

**Table:**

Refinement on *F*^2^	Hydrogen site location: inferred from neighbouring sites
Least-squares matrix: full	H-atom parameters constrained
*R*[*F*^2^ > 2σ(*F*^2^)] = 0.046	*w* = 1/[σ^2^(*F*_o_^2^) + (0.0731*P*)^2^ + 0.1699*P*] where *P* = (*F*_o_^2^ + 2*F*_c_^2^)/3
*wR*(*F*^2^) = 0.129	(Δ/σ)_max_ < 0.001
*S* = 1.07	Δρ_max_ = 0.27 e Å^−^^3^
3262 reflections	Δρ_min_ = −0.21 e Å^−^^3^
247 parameters	Extinction correction: *SHELXL2012* (Sheldrick, 2008), Fc^*^=kFc[1+0.001xFc^2^λ^3^/sin(2θ)]^-1/4^
0 restraints	Extinction coefficient: 0.0160 (14)
Primary atom site location: structure-invariant direct methods	

### Special details

**Table:**

Geometry. All esds (except the esd in the dihedral angle between two l.s. planes) are estimated using the full covariance matrix. The cell esds are taken into account individually in the estimation of esds in distances, angles and torsion angles; correlations between esds in cell parameters are only used when they are defined by crystal symmetry. An approximate (isotropic) treatment of cell esds is used for estimating esds involving l.s. planes.

### Fractional atomic coordinates and isotropic or equivalent isotropic displacement parameters (Å^2^)

**Table:**

	*x*	*y*	*z*	*U*_iso_*/*U*_eq_	
O1	0.1021 (2)	0.22514 (15)	0.69436 (8)	0.0438 (3)	
O2	0.42534 (16)	0.19900 (14)	0.42650 (7)	0.0318 (3)	
O3	0.5894 (2)	0.31428 (19)	1.03446 (9)	0.0505 (4)	
O4	0.8856 (2)	0.18161 (18)	1.00103 (10)	0.0521 (4)	
N1	0.2446 (2)	0.05926 (16)	0.60349 (8)	0.0288 (3)	
H1	0.3271	−0.0300	0.5983	0.035*	
N2	−0.10661 (19)	0.30173 (16)	0.44159 (8)	0.0285 (3)	
N3	0.08379 (19)	0.31651 (16)	0.39847 (8)	0.0273 (3)	
N4	0.6997 (2)	0.22341 (17)	0.99332 (9)	0.0342 (3)	
C1	0.2184 (2)	0.10192 (19)	0.68181 (10)	0.0295 (3)	
C2	0.3372 (3)	−0.02284 (19)	0.75387 (10)	0.0333 (4)	
H2A	0.2456	−0.0893	0.7878	0.040*	
H2B	0.4453	−0.0940	0.7277	0.040*	
C3	0.4314 (2)	0.04908 (18)	0.81419 (9)	0.0290 (3)	
C4	0.3122 (3)	0.1581 (2)	0.86039 (11)	0.0345 (4)	
H4	0.1727	0.1924	0.8516	0.041*	
C5	0.3993 (3)	0.2157 (2)	0.91904 (11)	0.0347 (4)	
H5	0.3196	0.2879	0.9502	0.042*	
C6	0.6072 (2)	0.16397 (18)	0.93061 (9)	0.0292 (3)	
C7	0.7300 (3)	0.0574 (2)	0.88551 (11)	0.0348 (4)	
H7	0.8698	0.0247	0.8940	0.042*	
C8	0.6403 (3)	0.0001 (2)	0.82728 (10)	0.0336 (4)	
H8	0.7208	−0.0721	0.7964	0.040*	
C9	0.1396 (2)	0.15810 (18)	0.53105 (9)	0.0273 (3)	
C10	−0.0656 (2)	0.20985 (19)	0.52365 (10)	0.0288 (3)	
C11	0.2410 (2)	0.22118 (18)	0.45045 (9)	0.0259 (3)	
C12	0.0955 (2)	0.35883 (18)	0.30627 (10)	0.0270 (3)	
C13	−0.0571 (3)	0.33649 (19)	0.25914 (10)	0.0321 (4)	
H13	−0.1676	0.2941	0.2878	0.039*	
C14	−0.0426 (3)	0.3779 (2)	0.16951 (11)	0.0377 (4)	
H14	−0.1457	0.3660	0.1378	0.045*	
C15	0.1246 (3)	0.4370 (2)	0.12657 (11)	0.0402 (4)	
H15	0.1350	0.4629	0.0662	0.048*	
C16	0.2765 (3)	0.4574 (2)	0.17385 (11)	0.0377 (4)	
H16	0.3896	0.4955	0.1449	0.045*	
C17	0.2615 (2)	0.42151 (19)	0.26367 (10)	0.0319 (4)	
H17	0.3612	0.4391	0.2951	0.038*	
C18	−0.2656 (2)	0.4522 (2)	0.42912 (11)	0.0346 (4)	
H18A	−0.2460	0.5174	0.4677	0.052*	
H18B	−0.3983	0.4248	0.4414	0.052*	
H18C	−0.2561	0.5122	0.3702	0.052*	
C19	−0.2353 (3)	0.1816 (2)	0.58962 (11)	0.0402 (4)	
H19A	−0.2664	0.2676	0.6206	0.060*	
H19B	−0.1943	0.0792	0.6296	0.060*	
H19C	−0.3545	0.1799	0.5612	0.060*	

### Atomic displacement parameters (Å^2^)

**Table:**

	*U*^11^	*U*^22^	*U*^33^	*U*^12^	*U*^13^	*U*^23^
O1	0.0579 (8)	0.0399 (7)	0.0327 (6)	0.0048 (6)	−0.0132 (5)	−0.0154 (5)
O2	0.0265 (6)	0.0381 (6)	0.0309 (6)	−0.0061 (4)	−0.0018 (4)	−0.0081 (5)
O3	0.0527 (8)	0.0643 (9)	0.0441 (8)	−0.0095 (7)	−0.0063 (6)	−0.0315 (7)
O4	0.0444 (8)	0.0567 (8)	0.0606 (9)	−0.0034 (6)	−0.0248 (6)	−0.0207 (7)
N1	0.0320 (7)	0.0311 (7)	0.0243 (6)	−0.0055 (5)	−0.0044 (5)	−0.0077 (5)
N2	0.0241 (6)	0.0364 (7)	0.0266 (7)	−0.0075 (5)	−0.0009 (5)	−0.0088 (5)
N3	0.0253 (6)	0.0338 (7)	0.0244 (6)	−0.0067 (5)	−0.0013 (5)	−0.0088 (5)
N4	0.0423 (8)	0.0332 (7)	0.0280 (7)	−0.0087 (6)	−0.0102 (6)	−0.0044 (6)
C1	0.0351 (8)	0.0311 (8)	0.0252 (7)	−0.0097 (6)	−0.0051 (6)	−0.0075 (6)
C2	0.0468 (9)	0.0282 (8)	0.0269 (8)	−0.0086 (7)	−0.0076 (7)	−0.0068 (6)
C3	0.0401 (9)	0.0276 (7)	0.0197 (7)	−0.0090 (6)	−0.0050 (6)	−0.0023 (6)
C4	0.0303 (8)	0.0438 (9)	0.0318 (8)	−0.0058 (7)	−0.0037 (6)	−0.0135 (7)
C5	0.0375 (9)	0.0380 (9)	0.0307 (8)	−0.0035 (7)	−0.0029 (6)	−0.0147 (7)
C6	0.0371 (8)	0.0291 (8)	0.0226 (7)	−0.0099 (6)	−0.0064 (6)	−0.0032 (6)
C7	0.0322 (8)	0.0377 (9)	0.0328 (8)	−0.0025 (6)	−0.0080 (6)	−0.0065 (7)
C8	0.0402 (9)	0.0307 (8)	0.0284 (8)	−0.0003 (6)	−0.0045 (6)	−0.0088 (6)
C9	0.0318 (8)	0.0304 (8)	0.0237 (7)	−0.0098 (6)	−0.0041 (6)	−0.0095 (6)
C10	0.0322 (8)	0.0337 (8)	0.0252 (7)	−0.0118 (6)	−0.0017 (6)	−0.0107 (6)
C11	0.0284 (7)	0.0275 (7)	0.0254 (7)	−0.0068 (6)	−0.0041 (6)	−0.0106 (6)
C12	0.0324 (8)	0.0251 (7)	0.0242 (7)	−0.0042 (6)	−0.0034 (6)	−0.0078 (5)
C13	0.0356 (8)	0.0319 (8)	0.0310 (8)	−0.0077 (6)	−0.0064 (6)	−0.0085 (6)
C14	0.0478 (10)	0.0357 (9)	0.0313 (9)	−0.0048 (7)	−0.0133 (7)	−0.0098 (7)
C15	0.0567 (11)	0.0367 (9)	0.0234 (8)	−0.0037 (8)	−0.0041 (7)	−0.0041 (6)
C16	0.0453 (10)	0.0329 (8)	0.0311 (9)	−0.0073 (7)	0.0037 (7)	−0.0023 (7)
C17	0.0345 (8)	0.0309 (8)	0.0306 (8)	−0.0071 (6)	−0.0029 (6)	−0.0064 (6)
C18	0.0310 (8)	0.0354 (8)	0.0390 (9)	−0.0041 (6)	−0.0019 (6)	−0.0137 (7)
C19	0.0346 (9)	0.0571 (11)	0.0315 (9)	−0.0166 (8)	0.0025 (7)	−0.0098 (8)

### Geometric parameters (Å, º)

**Table:**

O1—C1	1.217 (2)	C7—H7	0.9300
O2—C11	1.2436 (19)	C7—C8	1.384 (2)
O3—N4	1.2168 (19)	C8—H8	0.9300
O4—N4	1.2278 (19)	C9—C10	1.357 (2)
N1—H1	0.8600	C9—C11	1.435 (2)
N1—C1	1.3630 (19)	C10—C19	1.489 (2)
N1—C9	1.405 (2)	C12—C13	1.396 (2)
N2—N3	1.4047 (17)	C12—C17	1.390 (2)
N2—C10	1.373 (2)	C13—H13	0.9300
N2—C18	1.473 (2)	C13—C14	1.382 (2)
N3—C11	1.3905 (19)	C14—H14	0.9300
N3—C12	1.4206 (19)	C14—C15	1.386 (3)
N4—C6	1.463 (2)	C15—H15	0.9300
C1—C2	1.523 (2)	C15—C16	1.386 (3)
C2—H2A	0.9700	C16—H16	0.9300
C2—H2B	0.9700	C16—C17	1.384 (2)
C2—C3	1.508 (2)	C17—H17	0.9300
C3—C4	1.394 (2)	C18—H18A	0.9600
C3—C8	1.391 (2)	C18—H18B	0.9600
C4—H4	0.9300	C18—H18C	0.9600
C4—C5	1.381 (2)	C19—H19A	0.9600
C5—H5	0.9300	C19—H19B	0.9600
C5—C6	1.382 (2)	C19—H19C	0.9600
C6—C7	1.378 (2)		
			
C1—N1—H1	119.3	N1—C9—C11	123.26 (13)
C1—N1—C9	121.36 (13)	C10—C9—N1	127.98 (14)
C9—N1—H1	119.3	C10—C9—C11	108.77 (13)
N3—N2—C18	115.56 (12)	N2—C10—C19	120.59 (14)
C10—N2—N3	106.45 (12)	C9—C10—N2	109.94 (13)
C10—N2—C18	120.24 (13)	C9—C10—C19	129.46 (15)
N2—N3—C12	118.84 (12)	O2—C11—N3	124.28 (14)
C11—N3—N2	109.85 (12)	O2—C11—C9	131.03 (14)
C11—N3—C12	125.33 (12)	N3—C11—C9	104.68 (13)
O3—N4—O4	122.92 (14)	C13—C12—N3	120.30 (14)
O3—N4—C6	118.50 (14)	C17—C12—N3	119.20 (14)
O4—N4—C6	118.56 (14)	C17—C12—C13	120.49 (15)
O1—C1—N1	123.02 (14)	C12—C13—H13	120.3
O1—C1—C2	122.67 (14)	C14—C13—C12	119.43 (16)
N1—C1—C2	114.22 (13)	C14—C13—H13	120.3
C1—C2—H2A	108.6	C13—C14—H14	119.8
C1—C2—H2B	108.6	C13—C14—C15	120.40 (16)
H2A—C2—H2B	107.6	C15—C14—H14	119.8
C3—C2—C1	114.64 (13)	C14—C15—H15	120.1
C3—C2—H2A	108.6	C14—C15—C16	119.79 (15)
C3—C2—H2B	108.6	C16—C15—H15	120.1
C4—C3—C2	121.41 (15)	C15—C16—H16	119.7
C8—C3—C2	119.46 (14)	C17—C16—C15	120.67 (16)
C8—C3—C4	119.05 (14)	C17—C16—H16	119.7
C3—C4—H4	119.7	C12—C17—H17	120.4
C5—C4—C3	120.64 (15)	C16—C17—C12	119.16 (15)
C5—C4—H4	119.7	C16—C17—H17	120.4
C4—C5—H5	120.6	N2—C18—H18A	109.5
C4—C5—C6	118.71 (15)	N2—C18—H18B	109.5
C6—C5—H5	120.6	N2—C18—H18C	109.5
C5—C6—N4	118.90 (14)	H18A—C18—H18B	109.5
C7—C6—N4	118.88 (14)	H18A—C18—H18C	109.5
C7—C6—C5	122.21 (14)	H18B—C18—H18C	109.5
C6—C7—H7	120.8	C10—C19—H19A	109.5
C6—C7—C8	118.36 (15)	C10—C19—H19B	109.5
C8—C7—H7	120.8	C10—C19—H19C	109.5
C3—C8—H8	119.5	H19A—C19—H19B	109.5
C7—C8—C3	121.01 (15)	H19A—C19—H19C	109.5
C7—C8—H8	119.5	H19B—C19—H19C	109.5
			
O1—C1—C2—C3	−43.2 (2)	C4—C5—C6—N4	−179.40 (14)
O3—N4—C6—C5	1.4 (2)	C4—C5—C6—C7	0.1 (3)
O3—N4—C6—C7	−178.19 (15)	C5—C6—C7—C8	−0.5 (2)
O4—N4—C6—C5	−177.43 (15)	C6—C7—C8—C3	0.2 (2)
O4—N4—C6—C7	3.0 (2)	C8—C3—C4—C5	−0.7 (2)
N1—C1—C2—C3	140.27 (14)	C9—N1—C1—O1	1.0 (2)
N1—C9—C10—N2	−178.78 (14)	C9—N1—C1—C2	177.54 (13)
N1—C9—C10—C19	2.0 (3)	C10—N2—N3—C11	5.88 (16)
N1—C9—C11—O2	3.9 (2)	C10—N2—N3—C12	160.12 (13)
N1—C9—C11—N3	−177.63 (13)	C10—C9—C11—O2	−176.07 (15)
N2—N3—C11—O2	173.55 (13)	C10—C9—C11—N3	2.41 (16)
N2—N3—C11—C9	−5.07 (15)	C11—N3—C12—C13	130.08 (16)
N2—N3—C12—C13	−19.9 (2)	C11—N3—C12—C17	−49.4 (2)
N2—N3—C12—C17	160.63 (13)	C11—C9—C10—N2	1.18 (17)
N3—N2—C10—C9	−4.27 (16)	C11—C9—C10—C19	−178.02 (16)
N3—N2—C10—C19	175.01 (13)	C12—N3—C11—O2	21.4 (2)
N3—C12—C13—C14	−179.79 (14)	C12—N3—C11—C9	−157.26 (14)
N3—C12—C17—C16	177.76 (14)	C12—C13—C14—C15	1.7 (2)
N4—C6—C7—C8	179.08 (14)	C13—C12—C17—C16	−1.8 (2)
C1—N1—C9—C10	−56.9 (2)	C13—C14—C15—C16	−1.1 (3)
C1—N1—C9—C11	123.16 (16)	C14—C15—C16—C17	−0.9 (3)
C1—C2—C3—C4	57.6 (2)	C15—C16—C17—C12	2.4 (2)
C1—C2—C3—C8	−125.41 (16)	C17—C12—C13—C14	−0.3 (2)
C2—C3—C4—C5	176.33 (15)	C18—N2—N3—C11	142.19 (13)
C2—C3—C8—C7	−176.71 (15)	C18—N2—N3—C12	−63.56 (17)
C3—C4—C5—C6	0.4 (3)	C18—N2—C10—C9	−138.12 (14)
C4—C3—C8—C7	0.3 (2)	C18—N2—C10—C19	41.2 (2)

### Hydrogen-bond geometry (Å, º)

**Table:**

*D*—H···*A*	*D*—H	H···*A*	*D*···*A*	*D*—H···*A*
N1—H1···O2^i^	0.86	2.03	2.8658 (18)	164
C7—H7···O4^ii^	0.93	2.54	3.307 (2)	139
C18—H18*B*···O2^iii^	0.96	2.56	3.336 (2)	138

Symmetry codes: (i) −*x*+1, −*y*, −*z*+1; (ii) −*x*+2, −*y*, −*z*+2; (iii) *x*−1, *y*, *z*.
